# Factors associated with the use of antenatal care in Sindh province, Pakistan: A population-based study

**DOI:** 10.1371/journal.pone.0213987

**Published:** 2019-04-03

**Authors:** Jin-Won Noh, Young-mi Kim, Lena J. Lee, Nabeel Akram, Farhana Shahid, Young Dae Kwon, Jelle Stekelenburg

**Affiliations:** 1 Department of Healthcare Management, Eulji University, Seongnam, Korea; 2 Global Health Unit, Department of Health Sciences, University Medical Centre Groningen, University of Groningen, Groningen, the Netherlands; 3 Jhpiego, Johns Hopkins University, Baltimore, Maryland, United States of America; 4 National Institutes of Health Clinical Center, Bethesda, Maryland, United States of America; 5 Jhpiego, Karachi, Pakistan; 6 Department of Humanities and Social Medicine, College of Medicine and Catholic Institute for Healthcare Management, The Catholic University of Korea, Seoul, Korea; 7 Department of Obstetrics and Gynecology, Medical Centre Leeuwarden, Leeuwarden, the Netherlands; University of Campania, ITALY

## Abstract

**Background:**

Antenatal care (ANC) is critical to decrease maternal and neonatal mortality. However, little is known about the utilization of ANC services in Pakistan. This study assessed the utilization of ANC in Sindh province, Pakistan, and identified the factors that affect its use.

**Methods:**

We analysed a subset of data from Maternal and Child Health (MCH) Program Indicator Surveys conducted in Sindh province, Pakistan in 2013 and 2014. Respondents included 10,200 women who had given birth in the past two years. The outcome measure was making at least four ANC visits. Logistic regression models were used to identify demographic, socioeconomic, characteristics of ANC, and informational factors associated with ANC use.

**Results:**

Most women (83.5%) received one or more ANC, mostly by doctors (95%), but only 57.3% of them made the recommended four or more visits, and just 53.7% received their initial ANC care during the first trimester. Making four or more ANC visits was associated with: fewer household occupants (odds ratio [OR] = 0.98; 95% confidence interval [CI] = [0.97, 0.99]), large city residence (OR = 1.92; 95% CI = [1.57, 2.35]), higher women’s education (OR = 1.70; 95% CI = [1.33, 2.15]), greater household wealth (OR = 5.66; 95% CI = [4.22, 7.60]), and receiving MCH information from lady health worker (OR = 1.17; 95% CI = [1.00, 1.37]), mother-in-law (OR = 1.17; 95% CI = [1.01, 1.36]), other relatives/friends (OR = 1.19; 95% CI = [1.03, 1.38]), or nurse/midwife (OR = 1.31; 95% CI = [1.06, 1.61]).

**Conclusions:**

This study demonstrates that both socioeconomic factors and health information sources are associated with women’s use of ANC. Therefore, programs should target socially disadvantaged and vulnerable groups, particularly rural, less educated, and poor women, to improve utilization of ANC. In addition, strategies to increase exposure to MCH information sources should be a priority in Sindh, Pakistan.

## Introduction

Maternal mortality is one of the greatest health and development concerns worldwide, but especially in the developing world [1]. Complications throughout pregnancy, delivery and the postnatal period are the most common causes of death and disability among women of childbearing age [1,2]. Millions of women in developing countries do not have access to adequate healthcare services during pregnancy [2]. This fact alone is a major reason for poor health overall in the women [2, 3].

Antenatal care (ANC) is a key health service that can decrease maternal and neonatal mortality [2, 4]. For pregnant women with no perinatal complications, the World Health Organization (WHO) defined sufficient ANC as at least four healthcare visits during pregnancy. Recently, the 2016 WHO ANC model replaces four-visit focused model and recommends a minimum of eight healthcare provider contacts (up to 12 weeks, at 20 and 26 weeks of gestation, and at 30, 34, 36, 38 and 40 weeks) [2]. During ANC visits, pregnant women are educated about various danger signs and symptoms, which can significantly improve their own health and that of their infants during pregnancy, delivery, and the postpartum period [2, 3].

A growing body of literature has demonstrated a protective effect of ANC on maternal and child survival. According to Demographic and Health Surveys between 1990 and 2013 from 69 low-income and middle-income countries, at least one ANC visits reduced the probability of neonatal mortality by a 1.04% points and the probability of infant mortality by a 1.07%. Having recommended four or more ANC visits and at least once seen a skilled provider decreased the probability by an additional 0.56% and 0.42% points, respectively [4]. Several Indian studies have reported that ANC use increases the rate of institutional deliveries or home deliveries aided by skilled birth attendants [5, 6]. However, ANC use remains in low- and middle-income countries for several reasons, including poverty, low educational levels, and lack of access to a health facility [5–9].

In Pakistan, ANC is provided through the maternal and child health (MCH) services that are part of the existing primary healthcare system. The 2012–13 Pakistan Demographic and Health Survey (PDHS) revealed that 76% of women made at least one ANC visit during their last pregnancy (within five years of the survey). Furthermore, 73% of women received ANC from skilled providers (doctor, nurse, midwife, or lady health visitor [LHV]) during their last pregnancy. However, only 37% of women attended four or more ANC visits, while just 42% made their initial visit during the first trimester of pregnancy. The percentage of women whom received four or more ANC visits or early ANC initiation during their pregnancy varied by their place of residence, region/province, educational level, and household wealth index [10]. These findings justify further investigation into ANC services in Pakistan. However, few studies have focused on the quality of ANC in Pakistan, and little is known about the factors that influence its use. The purpose of this study was to assess the utilization of ANC in Sindh province, Pakistan, and identify the factors that affect utilization.

## Methods

### Data and subjects

We analysed a subset of data from the 2013 and 2014 MCH Program Indicator Surveys, which were conducted in the province of Sindh. Sindh has the highest total fertility rate in Pakistan at 4.3% [11]. The MCH Program Indicator Survey was created to monitor the implementation of maternal, newborn, and child health interventions as well as family planning and reproductive health interventions in Sindh [11]. The survey instrument was based on the PDHS questionnaire developed by Macro International, Inc. and the Knowledge, Practice and Coverage Survey questionnaire developed by the Johns Hopkins University/Child Survival Support Program 1990.

The survey employed a multi-stage, stratified sampling design using district-level population information. Districts are the third-order administrative divisions of Pakistan. Based on the most recent Census of Pakistan in 1998, a disproportionate sampling approach was used to allocate the sample to districts of rural and urban areas for better representation of smaller districts. Then a probability proportionate to size method was used to select cities and villages. A maximum of 10 participants were allocated to each village and 15–200 to each city selected to take part in the study. Ultimately, data were collected in all 23 districts of Sindh between June 2013 and October 2014 [11]. Trained interviewers visited each selected household. Study participants included married women age 15–49 who had a live birth in the two years prior to the survey and who resided in houses that were sampled for study participation. Only one participant was selected from each household. Each woman completed questionnaires about her last live birth. Data were collected from a total of 10,200 women (4,000 from the 2013 survey and 6,200 from the 2014 survey). A total of 10,200 were included with no missing variables in this analysis.

The female literacy rate is low in the Sindh Province of Pakistan. Therefore, female interviewers obtained informed consent verbally from each respondent, and then signed on behalf of the respondent. This study was approved by the Johns Hopkins University School of Public Health Internal Review Board (IRB00005002), and the National Bioethics Committee of Pakistan.

### Variables and measurement

The primary outcome variable was ANC utilization, which was defined as attending at least four ANC visits, as recommended by WHO four-visit focused model [2] because the data were obtained before establishing the 2016 WHO ANC model. To assess ANC use, respondents were asked: “How many times did you receive ANC during this pregnancy?” ANC referred to any pregnancy-related services provided by skilled health personnel, including doctors, nurse, LHV, and trained community midwives.

We reviewed previous literature [12] and the MCH Program Indicator Survey report [11] to identify factors that may be associated with ANC use and included them as independent variables in our study. They included two demographic factors (woman’s age and the number of household occupants), four socioeconomic factors (residence, women’s education level, husband’s education level, and the household wealth index), and MCH information sources. The wealth index was calculated using principal components analysis based on household assets [10]. Principal component analysis is a well-known statistical method to reduce dimensionality [13]. It was used to assess household wealth based on the value of 35 household assets. This index was then classified into quintiles.

To assess MCH information sources, respondents were asked, “During the last 12 months have you received any information about MCH from the following sources?” Possible responses included lady health worker (LHW), mother-in-law, other relatives/friends, Dai-traditional birth attendant (TBA), LHV, nurse/midwife, doctor, and media (radio, television, telephone helpline, text messages on mobile phone, health education/awareness session, print media). Binary variables (yes/no) were included for each response in the MCH information source section.

The characteristics of ANC included information on ANC use, number of ANC visits, type of ANC provider, place of ANC provision, timing of first ANC visit, and physical and laboratory examination received. The type of ANC provider is a multiple response question by asking, “Whom did you see?” Possible responses consist of LHW/Dai-TBA, nurse/midwife, and doctor.

### Statistical analysis

Descriptive statistics appropriate for the level of measurements were computed for all demographic and socioeconomic factors, MCH information sources, and characteristics of ANC variables. The ANC attendance was dichotomized as at least four times ANC visits (≥ 4, utilization, coded as “1”) and less than four times attendance (1–3, underutilization, coded as “0”) in binary logistic regression analyses, not including no ANC visit. Unadjusted model was run for demographic factors (woman’s age, number of household occupants), socioeconomic factors (residence, woman’s education level, husband’s education level, household wealth index), place of ANC, type of ANC provider, MCH information sources (LHW, mother-in-law, other relatives/friends, Dai-TBA, LHV, nurse/midwife, doctor, media), and survey year shown to be influential in the literature [12] using ANC utilization as the dependent variable. The unadjusted model showed significance for all demographic factors, socioeconomic factors, place of ANC, type of ANC provider (Dai-TBA, doctor), and MCH information sources. After controlling for all independent variables, the final multiple logistic regression was conducted. All analyses were performed using IBM SPSS software package version 25.0 (SPSS, Inc., IBM, Chicago, IL).

## Results

The mean age of the 10,200 respondents was 27.74 years (SD, 5.78 years), with a range of 15 to 49 years. The majority of women lived in rural areas (47.5%) and had no formal education (66.0%). Almost half of their husbands had no formal education (49.4%). Most women (83.5%, 8,521/10,200) received ANC during their last pregnancy. Among these women received any ANC, 42.7% of women had poor utilization of ANC during their pregnancy with once (10.9%), twice (16.5%), and three times (15.3%); more than half of women (57.3%) made the recommended four or more ANC visits. In addition, 53.1% of women made their initial ANC visit during the first trimester; 18.8% of the women did not initiated ANC until their third trimester. More women sought ANC from private health care facilities (69.6%) than public health care facilities (25.8%) or home (4.6%) (Table 1).

**Table 1:** Characteristics of survey respondents (n = 10,200).

Variables	Category		Mean (SD)/n (%)			
			Total	Underutilization (1–3) (n = 3,635)	Utilization (4+) (n = 4,886)	*p* value
*Demographic factors*					
Woman’s age (year), range		27.74 (5.78), 15–49	27.82 (6.05)	27.26 (5.33)	< 0.001
Number of household occupants, range		8.45 (4.63), 1–50	8.79 (4.81)	8.22 (4.55)	< 0.001
*Socioeconomic factors*					
Residence	Rural	4,846 (47.5)	2,147 (59.1)	1,386 (30.3)	< 0.001
	Town/small city	2,749 (27.0)	1,044 (28.7)	1,270 (27.8)	
	Large city	2,605 (25.5)	444 (12.2)	1,917 (41.9)	
Woman’s education level	No education	6,073 (66.0)	2,616 (74.8)	1,827 (48.5)	< 0.001
	Primary or middle	2,007 (21.8)	652 (18.6)	1,124 (29.8)	
	Secondary or higher	1,120 (12.2)	230 (6.6)	816 (21.7)	
Husband’s education level	No education	3,857 (49.4)	1,576 (53.3)	1,192 (38.5)	< 0.001
	Primary or middle	2,128 (27.3)	830 (28.1)	893 (28.9)	
	Secondary or higher	1,816 (23.3)	550 (18.6)	1,010 (32.6)	
Household wealth quintile	First (poorest)	2,040 (20.0)	921 (25.3)	328 (7.2)	< 0.001
	Second	2,040 (20.0)	1,003 (27.6)	530 (11.6)	
	Third	2,040 (20.0)	860 (23.7)	839 (18.3)	
	Fourth	2,040 (20.0)	547 (15.0)	1,290 (28.2)	
	Fifth (richest)	2,040 (20.0)	304 (8.4)	1,586 (34.7)	
*Characteristics of ANC*					
Timing of first ANC visit	First trimester	4,391 (53.1)	975 (27.2)	3,249 (72.3)	< 0.001
	Second trimester	2,320 (28.1)	1,232 (35.0)	1,029 (22.9)	
	Third trimester	1,557 (18.8)	1,315 (37.3)	213 (4.7)	
Place of ANC	Public care facility^a^	2,195 (25.8)	1,011 (27.8)	1,098 (24.0)	< 0.001
	Private care facility^b^	5,931 (69.6)	2,351 (64.7)	3,380 (73.9)	
	Home	394 (4.6)	273 (7.5)	95 (2.1)	
ANC provider (multiple responses allowed)					
LHW/Dai-TBA	No	8,109 (95.2)	3,351 (92.2)	4,461 (97.6)	< 0.001
	Yes	410 (4.8)	283 (7.8)	111 (2.4)	
Nurse/midwife	No	7,988 (93.7)	3,409 (93.8)	4,291 (93.8)	0.927
	Yes	549 (6.3)	226 (6.2)	282 (6.2)	
Doctor	No	461 (5.4)	311 (8.6)	124 (2.7)	< 0.001
	Yes	8,060 (94.6)	3,324 (91.4)	4,449 (97.3)	
*Mother and child health information sourc*e					
LHW	No	7,955 (78.0)	2,892 (79.6)	3,422 (74.8)	< 0.001
	Yes	2,245 (22.0)	743 (20.4)	1,151 (25.2)	
Mother-in-law	No	6,365 (62.4)	2,791 (76.8)	3,123 (68.3)	< 0.001
	Yes	3,835 (37.6)	844 (23.2)	1,450 (31.7)	
Other relatives/friends	No	6,365 (62.4)	2,497 (68.7)	2,578 (56.4)	< 0.001
	Yes	3,835 (37.6)	1,138 (31.3)	1,995 (43.6)	
Dai-TBA	No	8,440 (82.7)	2,971 (81.7)	3,956 (86.5)	< 0.001
	Yes	1,760 (17.3)	664 (18.3)	617 (13.5)	
LHV	No	9,298 (91.2)	3,371 (92.7)	4,059 (88.8)	< 0.001
	Yes	902 (8.8)	264 (7.3)	514 (11.2)	
Nurse/midwife	No	9,016 (88.4)	3,280 (90.2)	3,885 (85.0)	< 0.001
	Yes	1,184 (11.6)	355 (9.8)	688 (15.0)	
Doctor	No	5,866 (57.5)	2,229 (61.3)	2,228 (48.7)	< 0.001
	Yes	4,334 (42.5)	1,406 (38.7)	2,348 (51.3)	
Media^c^	No	7,863 (77.1)	2,993 (82.3)	3,160 (69.1)	< 0.001
	Yes	2,337 (22.9)	642 (17.7)	1,413 (30.9)	
*Survey*					
Year	2013	4,000 (39.2)	1,496 (41.2)	1,806 (39.5)	0.127
	2014	6,200 (60.8)	2,139 (58.8)	2,767 (60.5)	

Note: Numbers may not sum to total due to missing data.

ANC, antenatal care; LHV, lady health visitor, LHW, lady health worker; SD, standard deviation; TBA, traditional birth attendant

^a^ Includes government hospital, rural health clinics, basic health unit, dispensary, other public facilities

^b^ Includes private hospital/clinic, private doctor, homeopath clinic, dispenser/compounder, haki/dawakhana,other private facilities

^c^ Includes radio, television, telephone helpline, text message on mobile phone, health education/awareness session, print media

In the binary logistic regression, all independent variables were significant except for ANC from nurse/midwife and survey year. After controlling for the independent variables, most of the demographic and socioeconomic factors and information sources were significantly related to making at least the recommended four ANC visits compared to underutilization of ANC. The final model was significant, *x*^*2*^ (27, n = 5,458) = 1079.10, *p* < 0.001. The odds of making four or more ANC visits decreased significantly with the number of household occupants (odds ratio [OR] = 0.98; 95% confidence interval [CI] = [0.97, 0.99], *p =* 0.001), but not with women’s age. Women residing in large cities were significantly more likely to make the recommended number of ANC visits than rural residents (OR = 1.92; 95% CI = [1.57, 2.35], *p* < 0.001). While ANC use also increased significantly with women’s education, peaking among those with secondary or higher education (OR = 1.70; 95% CI = [1.33, 2.15], *p <* 0.001), there was no association with husband’s education. Wealth was the strongest determinant of ANC use: women in the top wealth quintile were six times more likely to make at least four ANC visits than women in the bottom quintile (OR = 5.66; 95% CI = [4.22, 7.60], *p <* 0.001). Women were significantly more likely to make the recommended number of ANC visits if they had received MCH information from a LHW (OR = 1.17; 95% CI = [1.00, 1.37], *p =* 0.049), mother-in-law (OR = 1.17; 95% CI = [1.00, 1.36], *p =* 0.043), other relatives/friend (OR = 1.19; 95% CI = [1.03, 1.38], *p =* 0.016), or nurse/midwife (OR = 1.31; 95% CI = [1.06, 1.61], *p =* 0.012) (Table 2).

**Table 2:** Logistic regression for making at least four antenatal care visits (n = 8,521).

		Adjusted	
		OR	95% CI
*Demographic factors*			
Woman’s age		1.00	(0.99, 1.01)
Number of household occupants		0.98	(0.97, 0.99)**
*Socioeconomic factors*			
Residence	Rural^a^	1.00	
	Town/small city	0.87	(0.74, 1.02)
	Large city	1.92	(1.57, 2.35)***
Woman’s education level	No education^a^	1.00	
	Primary or middle	1.33	(1.14, 1.55)***
	Secondary or higher	1.70	(1.33, 2.15)***
Husband's education level	No education^a^	1.00	
	Primary or middle	0.89	(0.77, 1.03)
	Secondary or higher	1.07	(0.91, 1.25)
Household wealth quintile	First (poorest)^a^	1.00	
	Second	1.31	(1.10, 1.59)**
	Third	2.03	(1.66, 2.49)***
	Fourth	3.53	(2.79, 4.50)***
	Fifth (richest)	5.66	(4.22, 7.60)***
*Characteristics of ANC*			
Place of ANC	Public^a^	1.00	
	Private	1.14	(1.00, 1.29)
ANC provider			
LHW/Dai-TBA		1.16	(0.70, 1.91)
Nurse/midwife		1.11	(0.70, 1.75)
Doctor		0.98	(0.75, 1.28)
*Mother and child health information source*			
LHW		1.17	(1.00, 1.37)*
Mother-in-law		1.17	(1.01, 1.36)*
Other relative/friends		1.19	(1.03, 1.38)*
Dai-TBA		0.91	(0.77, 1.08)
LHV		1.26	(0.99, 1.61)
Nurse/midwife		1.31	(1.06, 1.61)*
Doctor		1.14	(1.00, 1.30)
Media		0.99	(0.84, 1.16)
*Survey*			
Year	2013^a^	1.00	
	2014	0.98	(0.88, 1.14)

Note: Mother and child health information source and ANC provider coded as 0 = no, 1 = yes.

ANC, antenatal care; CI, confidence interval; LHV, lady health visitor; LHW, lady health worker; OR, odds ratio; TBA, traditional birth attendant

^a^ the reference category

*p < 0.05

**p < 0.01

***p < 0.001

## Discussion

Although a large majority of pregnant women in Pakistan (83.5%) received ANC, many did not meet all of the accepted standards for ANC [2, 4]. In this sample, only 57.3% of women made at least four visits and 53.1% attended in the first trimester (ideally before 12 weeks, but no later than 16 weeks). After controlling for all independent variables, appropriate ANC utilization (i.e., making at least four ANC visits) was significantly associated with smaller household size, large city residence, higher education for woman, greater household wealth, and MCH information from LHWs (trained personnel providing family planning and basic health services through home visits in rural areas), mother-in-law, other relatives/friends, or nurses/midwives.

Household size, measured as the number of persons in a particular household, was negatively associated with in the use of ANC in this study. This is consistent with previous research indicating that women with larger family sizes were less likely to utilize ANC due to excessive demand of their money, time and other resources [7, 9, 14–16]. In addition, women living in big cities were more likely to receive the recommended ANC visits than those in rural area in this study. This finding is consistent with previous studies, which found that urban women were more likely to use ANC than rural women [8, 10, 17–20]. This is not surprising since women living in a large city are better informed and have more access to health care. While rural women may depend on primary health care centers for MCH services, urban women have more options for ANC [18].

The more education women had, the more likely they were to make at least four ANC visits, confirming previous research on the impact of education on ANC utilization [8–11, 17, 21–24]. Educational level was also significantly associated with the timing of the first ANC check-up in Sindh [11]. One study has found that maternal education is a key factor that allows women to care about their ANC [25]. The impact of education is not limited to ANC: highly educated people engage in an array of healthy behaviors more often than less educated people. Education not only increases women’s awareness of the importance of health services, but also gives them the ability to select the most appropriate service for their needs [26, 27]. The impact of education is a particularly important issue for Pakistan, where only 11% of women have secondary or higher education. Our findings call for the establishment and expansion of health-promoting programs targeting less educated women to increase their awareness of the importance of ANC and enhance their use of it. In the long term, policies aimed at raising woman’s education may also increase the utilization of ANC services.

Several studies have found that a husband’s educational level is positively associated with adequate ANC use [8, 17, 24, 28]. In contrast, we found that the husband’s education was not significantly associated with making four or more ANC visits in Sindh. The sociocultural construct of masculinity in Pakistan offers a possible explanation. In joint families, a man is considered *besharam* (shameless) if he exhibits too great an interest in his pregnant wife. A belief system views pregnancy as a uniquely feminine attribute; therefore, men are excluded from their wives’ reproductive health issues, including ANC utilization [25].

Household wealth was strongly associated with ANC utilization. Women in the richest wealth quintile were approximately six times more likely to make the recommended number of ANC visits than women in the poorest wealth quintile. Previous studies have reported similar findings [7, 8, 10, 11, 16, 17, 22, 28]. Women from wealthier households are more likely to be able to afford routine health services like ANC and their associated costs, such as transportation, than are poor women [16]. More women received ANC in private health care facilities than public health care facilities. This may explain the high level of ANC utilization from private health care facilities among women who are living in urban areas with greater household wealth. It could be that educated and high socioeconomic class have better access to health care and have capacity to pay for health care and ANC visits and seek care from private sector because of their ability to pay that may affect ANC utilization.

The number and diversity of information sources associated with making the recommended number of ANC visits suggests that MCH information plays a vital role in determining ANC utilization. Few studies have explored the effect of MCH information on ANC utilization. Therefore, further research is needed to evaluate to what degree MCH information impacts women’s awareness of ANC in Pakistan, what messages and which sources have the greatest impact, and how they can be harnessed to promote ANC utilization. In this study, LHWs, nurse/midwife, mother-in-law, and relatives/friends who provided MCH information had a positive association with ANC use. In particular, women who received information from nurse/midwife among a skilled health provider were more likely to make a recommended ANC visits. The findings suggest that nurse/midwife play an important role by providing integrated preventive and curative health services, resulting in promoting ANC utilization in Pakistan. Several studies have found that women with high levels of exposure to mass media, such as television and radio, are more likely to receive ANC [8, 20, 29, 30]. In this study, however, the media were one of the few information sources that were not associated with ANC utilization. This discrepancy may be explained by the limited access to mass media in Pakistan and the severe restrictions on media freedom. In addition, media use in Pakistan–including mobile phones, the internet, and social media–is most common among men, young people, and urban residents [31]. Further research is needed to examine the effect of media-based MCH information on ANC utilization, after adjusting for demographic and socioeconomic determinants in Pakistan.

Several limitations of this study should be acknowledged. First, the study design was cross-sectional so that the associations found cannot necessarily be interpreted as causal relationships. The study used data from a single province, so the findings cannot be generalized to all of Pakistan. Second, women who had a live birth in the two years prior to the survey were included as study participants. Although trained interviewers assisted the participants using structured questions, respondents may under-report activities which are difficult to remember in detail because of the recall bias, the time lapse between childbirth and survey. Third, even though the study used the pretested and structured survey instrument, the independent variables were assessed at the time of the survey, not when the woman was pregnant and making decisions about ANC. It is possible that some of these variables, such as household wealth and MCH information source, changed after the birth of the child, which might lead to differences in interpretation and responses. Lastly, this study could not consider enough variables to see the utilization of ANC because of the data limitation. Therefore, further studies are needed to consider factors such as parity and place of birth.

## Conclusions

Our study found significant associations between socioeconomic factors and utilization of ANC services. These findings have demonstrated that efforts to improve ANC utilization should pay particular attention to the needs of rural, less educated, and poor women. Strategies to increase the accessibility and availability of health care service should be a priority in Sindh, Pakistan, particularly in rural areas. It is critical to develop health promotion programs that target women with low educational levels to enhance their awareness regarding the importance of ANC and increase their uptake of ANC services. At policy level, the study suggests that financial support that enables women from poor households reduce their out-of-pocket expenditure will have a positive effect on long-term ANC utilization. In addition, motivating women to receive ANC during their first trimester and recommended physical and laboratory examinations may help in enhancing the quality of ANC. As reflected by the results, there should also be strategies that emphasize on encourage women expose the MCH information sources to promote ANC utilization. Future research should investigate barriers to ANC utilization to inform appropriate interventions.

## Supporting information

S1 Dataset (XLSX)
